# A nationwide survey of public COPD knowledge and awareness in Saudi Arabia: A population-based survey of 15,000 adults

**DOI:** 10.1371/journal.pone.0287565

**Published:** 2023-07-05

**Authors:** Jaber S. Alqahtani, Abdulelah M. Aldhahir, Rayan A. Siraj, Abdullah A. Alqarni, Ibrahim A. AlDraiwiesh, Afrah F. AlAnazi, Areej H. Alamri, Roaa S. Bajahlan, Asalah A. Hakami, Saeed M. Alghamdi, Yousef S. Aldabayan, Abdullah S. Alsulayyim, Ahmed M. Al Rajeh, Saad M. AlRabeeah, Abdallah Y. Naser, Hassan Alwafi, Saeed Alqahtani, Ahmed M. Hjazi, Tope Oyelade, Mohammed D. AlAhmari

**Affiliations:** 1 Department of Respiratory Care, Prince Sultan Military College of Health Sciences, Dammam, Saudi Arabia; 2 Respiratory Therapy Department, Faculty of Applied Medical Sciences, Jazan University, Jazan, Saudi Arabia; 3 Respiratory Therapy Department, King Faisal University, Al-Ahsa, Saudi Arabia; 4 Department of Respiratory Therapy, Faculty of Medical Rehabilitation Sciences, King Abdulaziz University, Jeddah, Saudi Arabia; 5 Respiratory Care Program, College of Applied Medical Sciences, Umm Al-Qura University, Makkah, Saudi Arabia; 6 Department of Applied Pharmaceutical Sciences and Clinical Pharmacy, Faculty of Pharmacy, Isra University, Amman, Jordan; 7 Faculty of Medicine, Umm Al Qura University, Mecca, Saudi Arabia; 8 Department of Emergency Medical Services, Prince Sultan Military College of Health Sciences, Dammam, Saudi Arabia; 9 Department of Medical Laboratory Sciences, Prince Sattam Bin Abdulaziz University, Al-Kharj, Saudi Arabia; 10 UCL Division of Medicine, London, United Kingdom; Aga Khan University Karachi, PAKISTAN

## Abstract

**Background:**

There is a concerning lack of representative data on chronic obstructive pulmonary disease (COPD) awareness in Saudi Arabia, and a significant proportion of the population is vulnerable to developing a smoking habit, which is a major risk factor for the disease.

**Methods:**

Population-Based Survey of 15,000 people was conducted to assess the public knowledge and awareness of COPD across Saudi Arabia from October 2022 to March 2023.

**Results:**

A total of 15002 responders completed the survey, with a completion rate of 82%. The majority 10314 (69%) were 18–30 year and 6112 (41%) had high school education. The most common comorbidities among the responders were depression (7.67%); hypertension (6%); diabetes (5.77%) and Chronic Lung Disease (4.12%). The most common symptoms were dyspnea (17.80%); chest tightness (14.09%) and sputum (11.19%). Among those who complains of any symptoms, only 16.44% had consulted their doctor. Around 14.16% were diagnosed with a respiratory disease and only 15.56% had performed pulmonary function test (PFT). The prevalence of smoking history was 15.16%, in which current smokers were 9.09%. About 48% of smokers used cigarette, 25% used waterpipe and around 27% were E-cigarette users. About 77% of the total sample have never heard about COPD. Majority of current smokers (73.5%; 1002), ex-smokers (68%; 619), and non-smokers (77.9%; 9911) are unaware of COPD, p value <0.001. Seventy five percent (1028) of the current smokers and 70% (633) of the ex-smokers have never performed PFT, p value <0.001. Male, younger age (18–30 years), higher education, family history of respiratory diseases, previous diagnosis of respiratory disease, previous PFT, and being an ex-smokers increases the odds of COPD awareness, p-value <0.05.

**Conclusion:**

There is a significantly low awareness about COPD in Saudi Arabia, especially among smokers. A nationwide approach must include targeted public awareness campaigns, continued healthcare professional education, community-based activities encouraging diagnosis and early detection, advice on smoking cessation and lifestyle changes, as well as coordinated national COPD screening programs.

## Introduction

As global population ages and more people move into urban areas, chronic respiratory problems will remain a leading source of illness and death [1]. Worldwide, chronic obstructive pulmonary disease (COPD) is now recognized as a leading cause of mortality [2]. Identification of the risk factors of COPD is hindered by the substantial variance that exists across regions in terms of incidence, progression, and lung function trajectories at various ages [3]. Tobacco use and exposure to indoor air pollution (e.g., biomass burning), outdoor air pollution, and occupational pollutants have all been identified as major risk factors [4, 5]. Higher rates of hospital admissions, mortality, and morbidity attributable to COPD results in increased burden globally [6–8]. Inadequate medical expertise and low public awareness of the condition, limited availability of spirometry, and a lack of consensus on both clinical and epidemiological case definitions all contribute to missed diagnosis, misdiagnosis and misclassification of the disease [9, 10].

In Saudi Arabia, the incidence and prevalence of COPD have progressively increased between 1990 and 2019 [11]. In 2019, Alqahtani JS 2022 reported that approximately 434,560.64 individuals had COPD in Saudi Arabia, an increase of 329.82% from 1990 [vs 101,104.05] [11]. In 2019, COPD-related fatalities made up 57% of all deaths caused by chronic respiratory disorders in Saudi Arabia, accounting for around 2% of the total number of deaths in the country [11]. Although COPD has a significant impact on Saudi society, data and research on the awareness of COPD in the country is chronically limited. This lack of data is likely responsible for the severe underdiagnosis that has been observed [11]. Further, a recent survey of 385 people to assess public knowledge of the disease in one region of the kingdom showed that only 30% (116) of the respondents had prior awareness of COPD [12]. Health education on COPD is lacking, according to previous studies, not just among the general public but also those living with the illness and their loved ones [13, 14]. If comprehensive and efficient preventative actions are not taken, the rate of COPD incidence and prevalence will continue to rise [15, 16]. Therefore, awareness is essential to encourage prevention via smoking cessation advices, and may facilitate early detection, and individualize therapy. To the best of our knowledge, there is no representative sample-based research on public COPD awareness in Saudi Arabia. Therefore, the purpose of this study is to gauge public knowledge of COPD and its risk factors. This would aid in early diagnosis and guide targeted smoking cessation campaigns, with the goal of reducing the national burden of COPD on the heath system increasing the health-related quality of life of the populace.

## Methods

### Study design and study population

A cross-sectional study was conducted to assess public knowledge and awareness of COPD across Saudi Arabia from October 2022 to March 2023. Participants were recruited from the five major regions in the kingdom based on their suitability for the research. All participant willingly took part and gave their approval before beginning the questionnaire. The purpose and goals of the research were clearly outlined before the survey began. The inclusion criteria included all Saudis who are currently living in Saudi Arabia and are adults (aged ≥18 years).

Ethical approval for this study (IRB-2022-RC-01) was granted by the Institutional Review Board at Prince Sultan Military College of Health Sciences. Responding to the survey from the participants was considered as giving written consent. We did not include minors. The personal information was made anonymous and was scheduled to be deleted as soon as it was processed. The study was conducted in accordance with the Declaration of Helsinki.

### Survey tool and distribution

The four sections of this evaluation questionnaire were developed using data from the current literature and reviewed by a team of experts in respiratory medicine [17, 18]. The pilot version of the survey was administered to 15 participants including lay people and health care providers to assess face and content validity questions. Also, it determined an estimated completion time of five minutes for the questionnaire [19].

The survey was divided into four main sections, including structured and adaptive responses. The first section dealt with the characteristics of the participants, such as their gender, age, existing health conditions, family history of respiratory illness, and symptoms. The second section focused on their smoking habits. The third section evaluated their knowledge and understanding of COPD and where they obtained their information about the disease. The last component is made up of Likert scale questions (ranging from strongly disagree to strongly agree) that examine the participants’ perception, knowledge, acceptance, and expectations about COPD related risk.

For most Saudis, Arabic is the official language of communication. Because the survey’s original form was written in English, it was sent to the Professional Translation Unit at Prince Sultan Military College of Health Sciences to be translated into Arabic. Another professional translator specialist then translated the Arabic version back into English. Both experienced translators in the Translation Unit utilized "forward-backward translation" as suggested by the World Health Organization, to compare the two English versions [20]. A further pilot examination was carried out on the Arabic version, in which 10 individuals from the public were randomly involved, in order to assess the legibility and appropriateness of the questions [21].

The survey utilized a multi-channel approach to reach participants, including online channels such as social media and WhatsApp groups, as well as offline distribution in public places like shopping centers, cafes, and restaurants to also target individuals at risk of digital exclusion.

### Power calculation

With a confidence interval of 98%, a margin of error of 1%, and considering the adult population of Saudi Arabia in 2023 (27.58 million) and a response distribution of 50%, the minimum sample size was 13523 respondents.

### Statistical analysis

To summarize the characteristics of the respondents, a descriptive analysis was conducted. The comparisons between the different groups were assessed using Chi-square and Fisher’s exact tests. In addition, multiple logistic regression analysis was used to determine any variables associated with a lack of COPD knowledge. The data was processed using IBM SPSS 28 and a p-value < 0.05 considered statistically significant.

## Results

### Demographic characteristics

A total of 15002 participants completed this survey, with a completion rate of 82% (15002/18,292). The mostly represented regions are the south (35%) and central (23%) with the north (12%) being least represented. Most respondents were between 18 to 30 years of age (69%), had high school education (41%) and are females (64%)

### Comorbidities and symptoms of respiratory disease

The most common comorbidities among the responders were depression (7.7%); hypertension (6%); diabetic disease (5.8%); Anemia or other blood diseases (4.5%) and Chronic Lung Disease (4.1%). COPD, sleep-disordered breathing, and interstitial lung disease are all forms of chronic lung disease. The most common symptoms were dyspnea (17.8%); chest tightness (14.1%) and sputum (11.2%). Among those who complains of any symptoms, only 16.4% had consulted their doctor. Around 14.2% were diagnosed with a respiratory disease and only 15.6% had performed pulmonary function test (PFT) (Table 1).

**Table 1:** Demographics data and characteristics of the respondents (n = 15,002).

Variable	N (%)
**Gender**	
Male	5626 (37.50)
Female	9376 (63.50)
**What is your age range (year)**	
18–30	10314 (69)
31–40	2411 (16)
41–50	1325 (9)
51–60	652 (4)
> 60	300 (2)
**Highest education qualification**	
Elementary school	267 (2)
Intermediate School	948 (6)
High School	6112 (41)
Diploma	1719 (11)
Bachelors	5123 (34)
Masters	567 (4)
PhD	266 (2)
**Region**	
Eastern	2282 (15)
Western	2325 (16)
Central	3420 (23)
Northern	1744 (12)
Southern	5231 (35)
**Comorbidities**	
Depression	1150 (7.67)
High blood pressure	882 (6)
Diabetic disease	866 (5.77)
Anemia or other blood diseases	677 (4.51)
Chronic Lung disease	618 (4.12)
Stomach disease	575 (3.83)
Cardiac disease	497 (3.31)
Kidney disease	389 (2.60)
Osteoarthritis	378 (2.52)
Liver disease	188 (1.25)
Cancer	102 (0.68)
**Family history of respiratory disease**	
Yes	5692 (38)
No	9310 (62)
**Presence of any respiratory disease symptoms**	
No symptoms	9851 (65.66)
Dyspnea	2670 (17.80)
Chest tightness	2114 (14.09)
Sputum	1679 (11.19)
Wheezing	1231 (8.21)
Chronic cough	1019 (6.79)
**If subject complains of any symptoms: Have you consulted your doctor?**	
Yes	2467 (16.44)
No	3990 (26.60)
**Have you diagnosed with a respiratory disease?**	
Yes	2124 (14.16)
No	12878 (85.84)
**Have you ever had a pulmonary function test performed?**	
Yes (one time)	1435 (9.57)
Yes (several time)	901 (6.01)
No	12666 (84.42)

### COPD awareness

Almost 77% of the total sample have never heard about COPD. For those who heard about COPD, social media (42.02%); health care providers and School/university (36.10%) were the most common routes. The proportion of the population familiar with COPD increases with education level, in which COPD is known to 20% of individuals with a high school education or less, 20% of those with a bachelor’s, 26% of those with a master’s, and 38% of those with a PhD. COPD was unknown to 80% of individuals in the central region, 77% of people in the eastern region, 79% of people in the northern region, 73% of people in the southern region, and 80% of people in the western region (Table 2).

**Table 2:** Awareness of smoking as a risk factor for COPD.

Variable	N (%) or mean ± SD
**Smoking status**	
Current smoker	1364 (9.09)
Ex-smoker	910 (6.07)
Never smoked	12728 (84.84)
**If current or ex-smoker, which one of the following do you smoke?**	
Cigarette	1096 (48.01)
Water pipe (Shisha)	574 (25.14)
Vape or e-cigarette	613 (26.85)
**Pack per year For Cigarettes**	24.64 ± 40
**Number of Water pipe (20 min session) per week**	6 ± 6.3
**Do you believe that smoking is the main reason for COPD?**	
Yes	10776 (71.83)
No	1624 (10.83)
I don’t know	2602 (17.34)
**Does Tobacco cessation therapy have a significant role in the management of COPD?**	
Yes	11309 (75.83)
No	1255 (8.37)
I don’t know	2438 (16.25)
**Does the knowledge of COPD encourage you to stop or (not starting) smoking?**	
Yes	12269 (81.78)
No	2733 (18.22)
**Have you ever heard about COPD?**	
Yes	3470 (23.13)
No	11532 (76.87)
**If yes from, where have you heard about it?**	
Health care provide	1369 (39.32)
Friend	1108 (31.82)
TV or official health programs	810 (23.26)
Social media	1463 (42.02)
School/university	1257 (36.10)
Others	131 (3.76)

### Smoking status and COPD risk awareness

The prevalence of smoking history was 15.2%, of which current smokers were 9.1%. About 48% of the current or previous smokers smoked cigarette, 27% and 25% smoked e-cigarette and water pipe respectively (Table 2). Further, 45% of the current smokers, 37% of the former smokers, and 26% of people who never smoked were unaware that smoking is the leading cause of COPD, P value <0.001. Majority of current smokers (73.5%), ex-smokers (68%), and non-smokers (77.9%) were unaware of COPD as a disease, p value <0.001. Indeed, 24% of current smokers and 21% of ex-smokers said they were not motivated to quit by the awareness of COPD, p value <0.001.

Also, 75% (1028) of current smokers and 70% (633) of ex-smokers have never performed PFT. More than half of the all respondents (55.5%) diagnosed with respiratory disease have never performed PFT. Also, the prevalence of respiratory diseases was 24% among cigarette smokers, 22% among water pipe smokers, and 23% among e-cigarette users. Among those who felt unwell enough to see a doctor, 53% were diagnosed with a respiratory disease.

### General understanding of COPD epidemiology and prognosis

Table 3 shows the level of understanding about COPD as a disease of significant burden. The proportion of agreement (agree and disagree) was as follows: COPD is a common (48%), increases mortality (41.3%), COPD is a chronic disease (54%), COPD is a preventable disease (73.1%) and COPD is a treatable disease (60%). In the knowledge, acceptance and expectation parts, the total level of agreement was below 70% in all statements. This indicates poor public awareness.

**Table 3:** The general perception, knowledge, acceptance, and expectations about COPD among the study population.

Item	Level of agreement				
**Perception**	SA	A	N	D	SD
COPD is a very common disease	2954 (19.7)	4243 (28.3)	6101 (40.7)	1384 (9.2)	319 (2.1)
COPD has a severe mortality rate.	2080 (13.9)	4111 (27.4)	6862 (45.7)	1608 (10.7)	341 (2.3)
COPD is a chronic disease.	2797 (18.6)	5316 (35.4)	5733 (38.2)	866 (5.8)	290 (1.9)
COPD is a preventable disease.	4842 (32.3)	6127 (40.8)	3102 (20.7)	648 (4.3)	283 (1.9)
COPD is a treatable disease.	3019 (20)	6057 (40)	4504 (30)	1072 (7.1)	350 (2.3)
**Knowledge**					
COPD progresses with an exacerbation.	4813 (32)	5609 (37.4)	3800 (25.3)	525 (3.5)	255 (1.7)
This disease develops with the obstruction of the airways.	4066 (27)	5914 (39)	4146 (27.6)	584 (3.9)	292 (1.9)
The most important symptoms of COPD are shortness of breath, cough and sputum production.	4551 (30)	5887 (39)	3627 (24)	633 (4.2)	304 (2.1)
Symptoms of the COPD patients are more intense during the morning.	2797 (18.6)	3894 (26)	6363 (42.4)	1537 (10.2)	411 (2.7)
**Acceptance**					
Exposure of non-smokers to cigarette smoke in a smoking environment may cause COPD.	4358 (29)	5591 (37.3)	3862 (25.7)	882 (5.9)	309 (2.1)
Exposure to outdoor air pollution may cause COPD (especially fumes from vehicles exhaust in traffic).	4020 (26.8)	5908 (39.4)	3911 (26.1)	867 (5.8)	296 (2.0)
Exposure to organic and inorganic occupational dusts and chemicals may cause COPD	3949 (26.3)	6047 (40.3)	3820 (25.5)	833 (5.6)	353 (2.4)
Smoke from substances such as wood, dung, bushes, coal burned for heating and cooking at home may cause COPD.	3907 (26)	5562 (37.1)	4107 (27.4)	1086 (7.2)	340 (2.3)
**Expectation**					
Cessation of smoking may mostly prevent COPD.	3944 (26.3)	5763 (38.4)	3989 (26.6)	928 (6.2)	378 (2.5)
Some people may genetically develop COPD.	2697 (18)	4742 (31.7)	5112 (34.1)	1872 (12.5)	569 (3.8)
Influenza and pneumonia vaccines are needed to prevent from COPD.	3177 (21.2)	4967 (33.1)	5199 (34.7)	1211 (8.1)	448 (3.0)
Pulmonary rehabilitation is a recommended treatment option in COPD	3509 (23.4)	5726 (38.2)	4768 (31.8)	700 (4.7)	299 (2.0)
Avoid or controlling the risk factors after the development of COPD is necessary	5881 (39.2)	5075 (33.8)	3208 (21.4)	538 (3.6)	300 (2.0)

Abbreviations: SA: Strongly Agree; A: Agree; N: Neutral; D: Disagree; SD: Strongly Disagree

### Determinants of COPD awareness

Table 4 shows the factors associated with the level of knowledge about COPD. The level of COPD knowledge is significantly higher among 18–30 year olds compared to 31–40 age group [OR: 0.63 (0.57–0.72)], 41–50 age group [OR: 0.58 (0.49–0.76)], 51–60 age group [OR: 0.69 (0.56–0.84)], or ≥ 61 age group [OR: 0.74 (0.56–0.89)]. Living in the Eastern and Southern regions significantly associated with COPD level of awareness when compared to other regions, [OR: 1.21 (1.06–1.38)] and [OR: 1.63 (1.46–1.82)] respectively.

**Table 4:** Univariable and multivariable logistic regression models.

Descriptor	OR (95% CI)	Fully adjusted OR (95% CI)
**Gender**		
Female	**1**	**1**
Male	**1.11 (1.03**–**1.20)**	1.01 (0.93–1.11)
**Age**		
18–30 years	**1**	**1**
31–40 years	**0.79 (0.70–0.88)**	**0.63 (0.57–0.72)**
41–50 years	**0.76 (0.65–0.87)**	**0.58 (0.49–0.76)**
51–60 years	0.99 (0.82–1.19)	**0.69 (0.56–0.84)**
≥ 61 years	1.16 (0.89–1.50)	**0.74 (0.56–0.89)**
**Geographical region**		
Central	1	1
Eastern	**1.23 (1.08–1.40)**	**1.21 (1.06–1.38)**
Northern	1.11 (0.96–1.28)	1.09 (0.95–1.27)
Southern	**1.51 (1.36–1.67)**	**1.63 (1.46–1.82)**
Western	1.04 (0.91–1.19)	1.03 (0.90–1.18)
**Educational degree**		
Bachelor	1	1
Elementary school	1.04 (0.79–1.38)	0.87 (0.56–1.16)
Intermediate school	**0.69 (0.58–0.82)**	**0.64 (0.54–0.77)**
High school	**0.71 (0.65–0.77)**	**0.66 (0.60–0.72)**
Associate	**0.71 (0.62–0.81)**	**0.69 (0.60–0.79)**
Master’s	1.14 (0.94–1.39)	1.15 (0.98–1.45)
Doctoral	**1.68 (1.30–2.16)**	**1.67 (1.28–2.18)**
**Family history of respiratory diseases**		
No	1	
Yes	**1.52 (1.40**–**1.64)**	**1.45 (1.33**–**1.57)**
**Have respiratory disease**		
No	1	
Yes	**1.70 (1.54**–**1.88)**	**1.23 (1.10–1.38)**
**Had PFT done**		
No	1	
Once	**2.15 (1.92–2.42)**	**1.99 (1.76–2.25)**
More than once	**2.50 (2.16–2.86)**	**2.41 (2.07–2.80)**
**Smoking Status**		
Current	**1**	**1**
Ex-smokers	**1.30 (1.08–1.56)**	**1.25 (1.03–2.25)**
Never smoked	**0.78 (0.69–0.89)**	2.75 (0.71–10.64)
**Type of smoking**		
Never	**1**	**1**
Cigarette	**1.33 (1.16–1.53)**	3.04 (0.78–11.74)
Shisha	**1.48 (1.23–1.78)**	3.35 (0.86–12.92)
E-Cigarette	**1.52 (1.27–1.81)**	3.26 (0.83–12.68)

The multivariable logistic regression model was adjusted for gender, age, gender, and geographical region, and education, family history of respiratory disease, having had PFT, smoking status, and type of smoking.

Abbreviations: OR: odds ratio; PFT: pulmonary function testing

Level of education particularly bachelor’s degree is significantly correlated with COPD knowledge when compared to high school education [OR: 0.66 (0.60–0.72)], whereas it significantly increases further with doctoral degree [OR: 1.67 (1.28–2.18)]. Having a family history of respiratory illnesses is significantly associated with COPD awareness [OR: 1.45 (1.33–1.57)]. In addition, Performing PFT one time and more than once increases the likelihood to know about COPD by two-fold [OR: 1.99 (1.76–2.25) and OR: 2.41 (2.07–2.80), respectively]. Smoking cessation significantly increases the likelihood to familiarize with COPD by 25% [OR: 1.25 (1.03–2.25)], while never smoking significantly decreases the level of COPD awareness by 22% [OR: 0.78 (0.69–0.89)] when compared to current smoking.

## Discussion

To the best of our knowledge, this is the largest cross-sectional study that included 15002 subjects to investigate the level of public awareness of COPD and its relation to smoking in Saudi Arabia. Overall, our main outcomes indicate that there is a lack of COPD awareness and knowledge among the Saudi general population estimated around 77%, particularly among those who are currently (73.5%) and were previously (67%) smoking (*p* <0.001). This findings is consistent in both developed and developing countries worldwide [22]. Several studies have reported varying but low COPD awareness rates. The lowest was found in an Indian survey that included over 6000 participants from urban cities and reported 0.9% COPD awareness rate [23], whereas it was 4% in Brazil [22, 24], 8% in France [25], 10% in Germany [22], 17% in Spain [26] and Canada [27], and 21.3% in Japan [28]. Although the current study demonstrated relatively higher rate of COPD awareness (23.13%) in Saudi Arabia, there is still a significant gap of knowledge, particularly among those aged 41–50 years old [OR: 0.58 (0.49 to 0.76)] and 51–60 years old [OR: 0.69 (0.56 to 0.84)], despite being at a greater risk of developing COPD [29].

As a primary cause for COPD [30], the prevalence of smoking is estimated to be around 20% [31] and is continuously rising alongside COPD rates in Saudi Arabia [11, 32]. This can be attributed to the existing lack of knowledge either among smokers towards COPD or among the public regarding the relationship between respiratory illnesses and smoking. We found that over two third of current smokers and ex-smokers have no knowledge of COPD, with close to half of the current smokers and ex-smokers doubts that smoking is a leading cause of COPD. Two previous studies conducted by Mun et al. [33] and Seo et al. [34] have also shown significantly low COPD awareness rates among heavy smokers that participated in cigarette cessation programs in South Korea (0.4% and 1.0%, respectively). These show a chronic lack of COPD awareness among smokers. Although we found higher COPD awareness rates among smokers or ex-smokers in Saudi Arabia, creative new effort is needed to translate this awareness to increased smoking cessation.

Interestingly, 18% of the public and 24% of current smokers in this study showed lack of motivation to quit smoking despite being informed about COPD. This is higher than the 5.5% reported by Seo et al [34] as well as the 9% in a study by Carlos et al [35] conducted in the United States. A significantly high doubt that COPD is linked with smoking among the participants in this study (36%) may partly explain this low quitting intent. Perhaps, the current COPD public awareness and education approach needs systematic review and overhaul to reduce COPD burden in Saud Arabia in line with the national vision 2030 [36]. Interestingly, ex-smokers have significantly more likely to be informed about COPD [OR: 1.25 (1.03 to 2.25)] compared with current smokers, which suggests that knowledge and perception about COPD may play a vital role in smoking cessation. Thus, more could be invested into targeted COPD public education in-conjunction with broader smoking cessation programs.

COPD is often misdiagnosed and underestimated in Saudi Arabia [32, 37, 38] and early diagnosis of COPD could minimize both medical and economic burden of the disease [39]. Our study found that among those who showed signs and symptoms of respiratory distress, only around 16% had consulted their physician or performed PFT. While more than half (56%) of patients previously diagnosed with respiratory diseases had never been assessed by spirometry. Also, 75% of current smokers and 70% of former smokers, who are at a greater risk of developing COPD, have never performed PFT (p<0.001). This underutilization of spirometry was also observed in two studies conducted in Germany by Härtel et al [40] and Heinmüller et al. [41] that reported 27% and 29% spirometry prevalence rate among COPD patients, respectively. Also a previous study conducted in a primary care setting showed a prevalence of annual spirometry use of around 27% in patients with COPD in the United States [42]. Taken together, this low rate of PFT along with the existing lack of COPD awareness could substantially contribute to the current under-detection and under-treatment of COPD and may add further burden on health systems [41].

Expectedly, we found that COPD awareness tend to be 99% higher among participants who had a single spirometry and the awareness increased to 2.4 folds when more spirometry test was performed. Therefore, a key policy priority should be established to facilitate public access to primary health centers with pulmonary function laboratories and encourage high-risk population to routinely conduct self-assessment and medical examination.

The overall level of agreement towards most of the surveyed COPD-related statements was below 70%, which suggests insufficient COPD awareness and expectations among the public. More than half of the respondents were unaware that COPD is a common disease and nearly 60% of the participants did not know that COPD increases mortality, despite COPD being ranked as the third leading cause for mortality globally [11]. Although lower COPD acceptance rates were also reported in Australia [43] and India [44], this poor level of beliefs and perception is particularly higher in Saudi Arabia possibly due to social pressure since most Saudi youths socialize mostly in indoors and areas with high exposure to passive smoking [8]. Therefore, there is a paramount necessity to provide valid information to the Saudi public regarding the nature, prevalence, risk factors, and the long-term consequences of COPD including the alarming reduction in disability-adjusted life years (DALYs) [45]. However, we found a trend that COPD awareness may increases in parallel with the level of education, which highlights the need for increasing the efforts to expand the COPD educational campaigns targeting young as well as uneducated older adults. A similar awareness initiative showed promising results in a four-years Norwegian public campaign where COPD awareness level was raised from 27% to 78% [46]. Our results are likely to bring further intriguing data regarding COPD in Saudi Arabia, expanding on past studies in this field [47–51].

This population-based research of 15,000 people has both strengths and drawbacks. With a large sample size, this study could provide accurate prevalence rates and data about the level of awareness that are also typical of the population under investigation. Furthermore, they provide generalizability, statistical power, and insights into the attitudes and behaviors of the Saudi population towards COPD. However, sampling, response or recall bias are the main limitations of this study. It’s important for future research to take into account possible barriers to participation, such as respondents’ busy schedules, a lack of incentives, a lack of interest, or a complicated survey.

This nationwide assessment of public COPD knowledge and awareness in Saudi Arabia have numerous implications. From a research perspective, this study might give insights on the existing level of public awareness and understanding of COPD in Saudi Arabia. The findings may also help in identifying areas of emphasis for public health campaigns and educational initiatives aimed at increasing public knowledge and understanding of COPD. Clinically, it emphasizes the need for expanded, targeted COPD screening and diagnosis in Saudi Arabia, as well as improved disease management and therapy. Moreover, the results may aid healthcare practitioners in designing focused teaching programs and resources for their COPD patients. Overall, population-based surveys of 15,000 individuals have the potential to give valuable scientific and therapeutic insights, assisting in identifying areas for improvement and guiding public health and clinical interventions.

## Conclusion

There is a significant lack of awareness and knowledge of COPD among the general public in Saudi Arabia; this problem is more prevalent among smokers and people with lower education levels. This stresses the need for a multifaceted national strategy to improve Saudi Arabia’s low COPD awareness, including public awareness campaigns, healthcare professionals education, community-based initiatives focused on the importance of early detection, smoking cessation, and lifestyle modifications, as well as population screening for COPD.
